# Is aerial dispersal an overlooked pathway for ectomycorrhizal truffle fungi?

**DOI:** 10.1007/s00572-026-01296-x

**Published:** 2026-07-28

**Authors:** Peter G. Kennedy, Andrew W. Ratz, Marcos V. Caiafa, Heather A. Dawson, Carolyn A. Delevich, Bitty A. Roy, Matthew E. Smith

**Affiliations:** 1 Department of Plant & Microbial Biology, University of Minnesota, 1470 Gortner Avenue, Saint Paul, MN 55108 USA; 2 Departamento de Botânica, Universidade Federal de Santa Catarina, Florianópolis, SC Brazil; 3 Institute of Ecology and Evolution, University of Oregon, Eugene, OR 97402 USA; 4 Department of Plant Pathology, University of Florida, Gainesville, FL 32611 USA

**Keywords:** Mycorrhizal fungi, Hypogeous, Community assembly, Aerobiology, Mycophagy

## Abstract

The dispersal of ectomycorrhizal (EM) fungi is typically characterized by an ecological dichotomy in which aboveground (epigeous) mushrooms disperse via wind and belowground (hypogeous) truffles disperse via animal-mediated movement. To assess whether hypogeous EM fungi may be dispersed by wind more often than generally recognized, we used high-throughput sequencing of paired spore trap and soil samples at eight sites across the United States. Within the spore trap samples, hypogeous EM taxa represented 17.2 ± 1.62% (mean ± SE) of the total EM OTUs and 19.4 ± 1.75% of the total EM sequence reads. In general, their presence was characterized by episodic higher abundance events rather than continuous occupancy as compared to epigeous EM taxa. The hypogeous EM taxa found in spore traps also had significantly smaller average spore volumes than those remaining in the soil, likely facilitating their presence in air. Further, by identifying multiple instances of the same hypogeous EM OTU being present in spore trap and soil samples located tens of km apart, our study suggests wind may carry spores of hypogeous EM fungi considerable distances. Collectively, these findings indicate that the boundary between dispersal pathways for hypogeous EM fungi may be more fluid than previously thought and that specific traits and vectors allow them to exploit the atmosphere as a complementary dispersal pathway.

**Supplementary Information:**

The online version contains supplementary material available at 10.1007/s00572-026-01296-x.

## Introduction

Since the start of the molecular revolution (Horton and Bruns 2001), DNA-based identification techniques have allowed researchers to characterize mycorrhizal fungal communities in all parts of any ecosystem – i.e. in soil, on roots, as sporocarps, and in air (Peay et al. 2016). In particular, the use of high-throughput sequencing has allowed for increasingly robust ecological analyses, often based on hundreds of samples and millions of sequence reads (Bálint et al. 2016; Tedersoo et al. 2022). Although the progress in characterizing mycorrhizal fungal communities at the global scale has been impressive (Větrovský et al. 2020; van Galen et al. 2025), relative to other ecosystem compartments, the structure of mycorrhizal fungal communities in air has been less well documented (Chaudhary et al. 2022). Understanding this habitat from an ecological perspective is important, as the sexual and asexual spores used by mycorrhizal fungi to disperse represent the reproductive output of fungal populations as well as a basis for future community assembly (Womack et al. 2015; Aguilar-Trigueros et al. 2019).

Ectomycorrhizal (EM) fungi have evolved a wide variety of sporocarp morphologies from which their spores are released (Bruns et al. 1989; Varga et al. 2019). One of the most well recognized ecological dichotomies among EM sporocarps is their location, with many EM fungi producing sporocarps that emerge aboveground (referred to as epigeous) and a diverse subset of EM fungi that produce sporocarps that stay entirely belowground (referred to as hypogeous). Unlike epigeous sporocarps that rely primarily on wind for dispersal, hypogeous sporocarps are dependent upon consumption or disturbance by a variety of animals for dispersal (Frank et al. 2006; Maser et al. 2008; Elliott et al. 2022; Stephens et al. 2025). This is because hypogeous EM taxa make sporocarps (a.k.a. truffles), which have lost forcible spore discharge and are not directly exposed to wind due to their subterranean location. To attract animal dispersers, truffle-forming EM fungi often produce sporocarps with strong odors (Stephens et al. 2020) or produce truffles that visually match the shape and color of fruits (Caiafa et al. 2021). The morphological adaptation of producing hypogeous sporocarps appears to be evolutionarily successful, having evolved in nearly all major EM lineages (Tedersoo and Smith 2013), and is particularly common in seasonally dry habitats where belowground sporocarp production may prevent desiccation (Bruns et al. 1989).

Although EM fungi represent a smaller portion of the global aerial mycobiome than other guilds such as plant pathogens and wood saprotrophs (Abrego et al. 2024), they can often comprise a significant portion of the aerial community in latitudes where their hosts are abundant and phylogenetically diverse (Abrego et al. 2024; Ratz et al. 2026). For example, working in a temperate forest in New Hampshire, USA, Borgmann-Winter et al. (2023) showed that co-sampling of fungal propagules present in air and in mammal scat contained hundreds of EM amplicon sequence variants (ASVs). Notably, the authors showed that there was minimal overlap in the EM fungal communities in air and scat, with members of the aerial community dominated by epigeous taxa (e.g. *Tomentella*, *Inocybe*, *Ramaria*) whereas members of the scat community were dominated by a mix of other epigeous taxa (e.g. *Cortinarius* and *Russula*) and hypogeous taxa (e.g. *Elaphomyces* and *Melanogaster*). They also found that scat samples had a high abundance of arbuscular mycorrhizal (AM) fungal spores, which either produce hypogeous sporocarps or they lack sporocarps. Collectively, these results reinforce the widely accepted paradigm that hypogeous EM fungi are reliant on animal consumption for dispersal and are functionally absent from the aerial mycobiome.

While the general understanding of hypogeous propagule dispersal is centered on animal consumption and subsequent fecal deposition, recent work on AM fungi suggests that aerial dispersal may be more a common pathway than previously thought. Using air sampling devices located on a building rooftop in a major American city, Chaudhary et al. (2020) documented a diverse array of AM fungal taxa in air, with many taxa displaying unique seasonal abundance patterns. The researchers also showed that the AM fungal spores present in the air samples belonged disproportionally to species with smaller spore diameters, presumably because they are more likely to become and stay aerosolized. Along with being blown into air, the caching of hypogeous sporocarps in elevated locations may create another pathway for airborne hypogeous propagules (Maser et al. 2008). As reviewed in Elliott et al. (2022), many small mammals have been well documented to cache significant quantities of hypogeous sporocarps in tree canopies for later consumption. If portions of a sporocarp are dropped during consumption or a partially consumed sporocarp is left in an elevated position (directly exposing spores to wind), it is likely that spores would be released into surrounding air. Given these possible pathways, neither of which are mutually exclusive, the presence of hypogeous EM propagules in air may occur more often than previously recognized. However, repeated, standardized sampling across multiple sites and habitats would be necessary to quantify its ecological significance.

To assess the potential for hypogeous EM fungal spores to be part of the aerial EM mycobiome, we used a replicated sampling design spanning multiple sites and habitat types within North America. Specifically, we collected both spore trap and soil samples over a two-year period at eight sites across the United States and then characterized the EM fungal communities using high-throughput sequencing. Our overarching goal was to quantify the presence and abundance of hypogeous EM fungal spores in the atmosphere at both local and continental scales, using soil samples to assess potential trap contamination as well as to better understand possible distances associated with wind dispersal. In addition to documenting which hypogeous EM fungi were present in aerial samples, we tested two hypotheses about their presence in air: (H1) hypogeous EM taxa would be present in a limited number of samples, but, when present, would be in higher abundance than epigeous EM taxa, reflecting discrete ecological events facilitating their dispersal; (H2) hypogeous EM taxa with smaller spores would be overrepresented in the atmosphere compared to those with larger spores.

## Methods

### Study sites

**Fig. 1:**
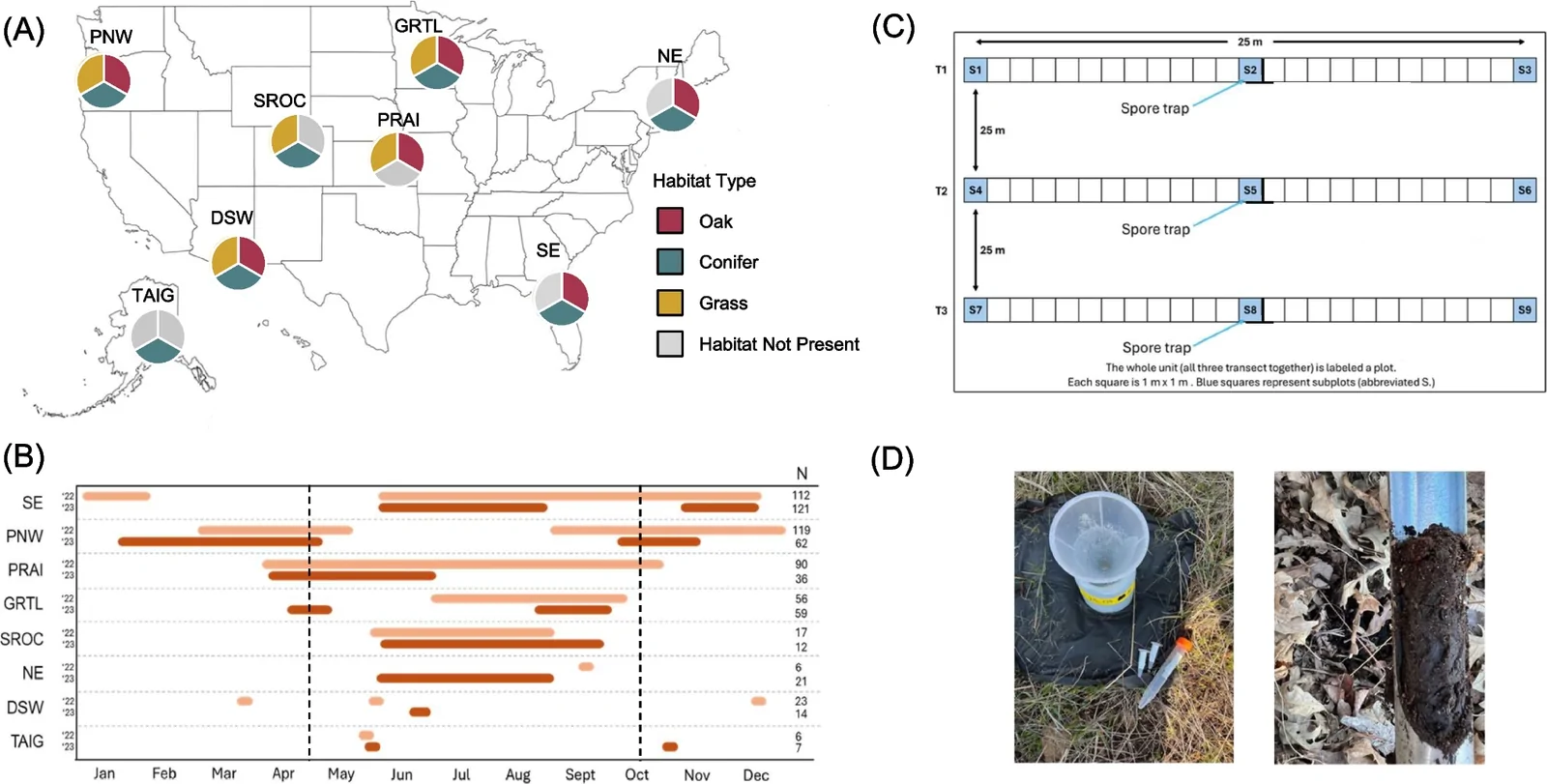
Sampling locations, times, and methods. (**A**) The locations of the eight sites sampled, with the habitat types present at each site. Burn treatments were pooled based on results of Ratz et al. (2026). Habitats were considered present if either a burned or unburned treatment of that habitat was present at that site. (**B**) The time periods over which spore traps were deployed at each site, with the dotted vertical lines being the dates of soil sampling. N = represents the number of spore trap samples per site per year (2022 or 2023). (**C**) The layout of the plots within each habitat type, with three 25 m transects and spore traps located in the middle of each transect (S2, S5, and S8). (**D**) A spore trap deployed in a grassland habitat and soil sample taken from an unburned oak forest habitat

We sampled 31 plots across eight sites in the United States, primarily utilizing sites from the National Ecological Observation Network (NEON) and National Science Foundation Long-term Ecological Research (LTER) networks (Fig. 1). Plots within the eight sites represented three habitats; 1) grasslands, 2) Pinaceae-dominated conifer forests, and 3) oak-dominated deciduous forests, which were separated by distances ranging from 100 m to 60 km. Habitats were originally separated as burned and unburned plots (Fig. 1), although the burn treatments were pooled at the site level because they were previously shown to not significantly differ in their aerial macrofungal communities (Ratz et al. 2026). Due to regional availability, not all habitat combinations were represented at every site. Further description of dominant vegetation and climatic conditions can be found in the supplementary information (Table S1).

### Spore sampling

Each plot contained three parallel 25 m transects, spaced 25 m apart, with a modified passive spore trap at each center (subplots 2, 5, and 8, Fig. 1). Traps, which had an opening height of 15 cm above the soil surface, used a 10 cm diameter funnel with 500 μm mesh to exclude insects and debris that drained into a 1 L bottle containing 100% ethanol to prevent microbial growth. To minimize soil splash contamination, a 36 cm^2^ patch of landscape fabric was placed under each trap. Traps were collected weekly to tri-monthly (depending on rainfall) over two years (starting Spring 2022 and ending Fall 2023), with intervals adjusted based on rainfall (median = 27 days). In the first year, sampling was done as often as the field season or available workers allowed, while in the second year, deployment was specifically targeted to coincide with peak spring and fall epigeous fungal fruiting identified via iNaturalist phenology data (Figure S1).

### Soil sampling

Soil cores were sampled at nine subplots per site in Spring 2022, but then reduced to five subplots (1, 2, 5, 8, and 9) for Fall 2022, Spring 2023, and Fall 2023. Samples were collected on four specific dates: May 1, 2022; October 23, 2022; May 21, 2023; and October 24, 2023. Litter was removed from the surface, followed by a ~ 10 cm soil core using a 2.54 cm sampler. Tools were sterilized with ethanol between samples, and all materials were kept frozen at −20 °C until processing.

### Molecular identification

Filter surfaces from spore traps were scraped into 2 ml centrifuge tubes with flame-sterilized tools and DNA was extracted from all sample types using the Qiagen DNeasy PowerSoil Pro Kit. The ITS1 region was amplified using ITS1F and ITS2 primers (Smith and Peay 2014). PCR was conducted in two rounds: an initial 30-cycle amplification followed by a 12-cycle round to attach dual index barcodes. Products were purified, quantified, and pooled at equimolar concentrations. The spore trap and soil libraries were sequenced at separate facilities (spore trap – University of Minnesota, soil – University of Oregon) using Illumina MiSeq or NextSeq (2 × 300 bp) runs with a 10% PhiX spike-in.

### Bioinformatics

All sequences from both sample types were concurrently processed in the same bioinformatics pipeline. Using cutadapt (Martin 2011) and vsearch (Rognes et al. 2016), sequence reads were filtered for quality, trimmed of primers, merged, and filtered for lengths between 100–500 bp. Denoising was performed via UNOISE3 (Edgar 2016) to produce amplicon sequence variants (ASVs), which were then screened for chimeras and clustered into operational taxonomic units (OTUs) at 98% identity. Taxonomy was assigned using the SINTAX algorithm against the EUKARYOME v. 1.9.4 database (Tedersoo et al. 2024). Sequencing depth ranged from 1 to 83,853 reads within the soil samples and ranged from 734 to 288,013 within spore samples with medians of 22,824 and 127,697 respectively. Given the high variation in sequencing depth across samples, all were rarefied to 1,000 reads per sample. To preserve the biological presence of rare taxa, OTUs appearing as singletons within a sample post-rarefaction were retained. Because the data underwent error-correction and clustering prior to subsampling, these singletons represent high-confidence variants rather than sequencing artifacts. Using the FungalTraits database (Põlme et al. 2020), OTUs were classified as ectomycorrhizal (EM) or not. All EM taxa were further classified as hypogeous or not using literature and author knowledge (Table S2). Further, for all hypogeous EM OTUs captured in either spore traps or seen in soil samples, spore volumes were determined via literature review (Table S3). Raw.fastq sequence files were deposited in the National Center for Biotechnology Information (NCBI) Short Read Archive (SRA) under BioProject numbers PRJNA1291903 (spore trap) and PRJNA1327291 (soil).

### Statistical analyses

All statistical analyses were conducted in RStudio (R version 4.3.3, RStudio release September 2024). The abundance, based on the sum of read counts within samples, and richness of EM fungal OTUs across habitats were compared using ANOVAs, with Tukey HSD post-hoc tests to determine significant differences among means. Abundance-occupancy relationships for hypogeous and epigeous fungi were assessed using linear models and then compared with one another using nested ANOVAs along with AIC selection to determine p-value and confirm model selection, respectively. The correlation between hypogeous and epigeous EM fungal abundance and richness was tested using both linear models and a generalized additive model. EM fungal spore volumes were compared between spore trap and soil samples using t-tests. Finally, variation in spore trap filter particulate loads was analyzed using linear models alongside ANOVAs.

## Results

After processing, there were a total of 622 spore trap and 757 soil samples across 8 sites, with 374 spore samples and 630 soil samples containing EM reads. The spore trap and soil samples had a total of 2,019 and 11,950 EM OTUs from 5,903 and 185,052 total sequence reads representing 89 and 103 distinct genera, respectively. The spore trap and soil samples contained 1,341 (66%) and 4,423 (37%) EM OTU singletons respectively. As expected, both datasets had higher mean OTU richness and proportional abundances of EM fungal sequence reads in the two forest habitats (Pinaceae-dominated forests and oak forests, where host density and richness were much higher) than in the grassland habitat (Figure S2). Within the spore trap samples, hypogeous EM taxa represented 17.2 ± 31.3% of the total EM OTUs and 19.4 ± 33.9% of the total EM sequence reads, while in the soil samples, hypogeous EM taxa represented 12.3 ± 18.2% of the total EM OTUs and 11.5 ± 20.9% of the total EM sequence reads.

**Fig. 2:**
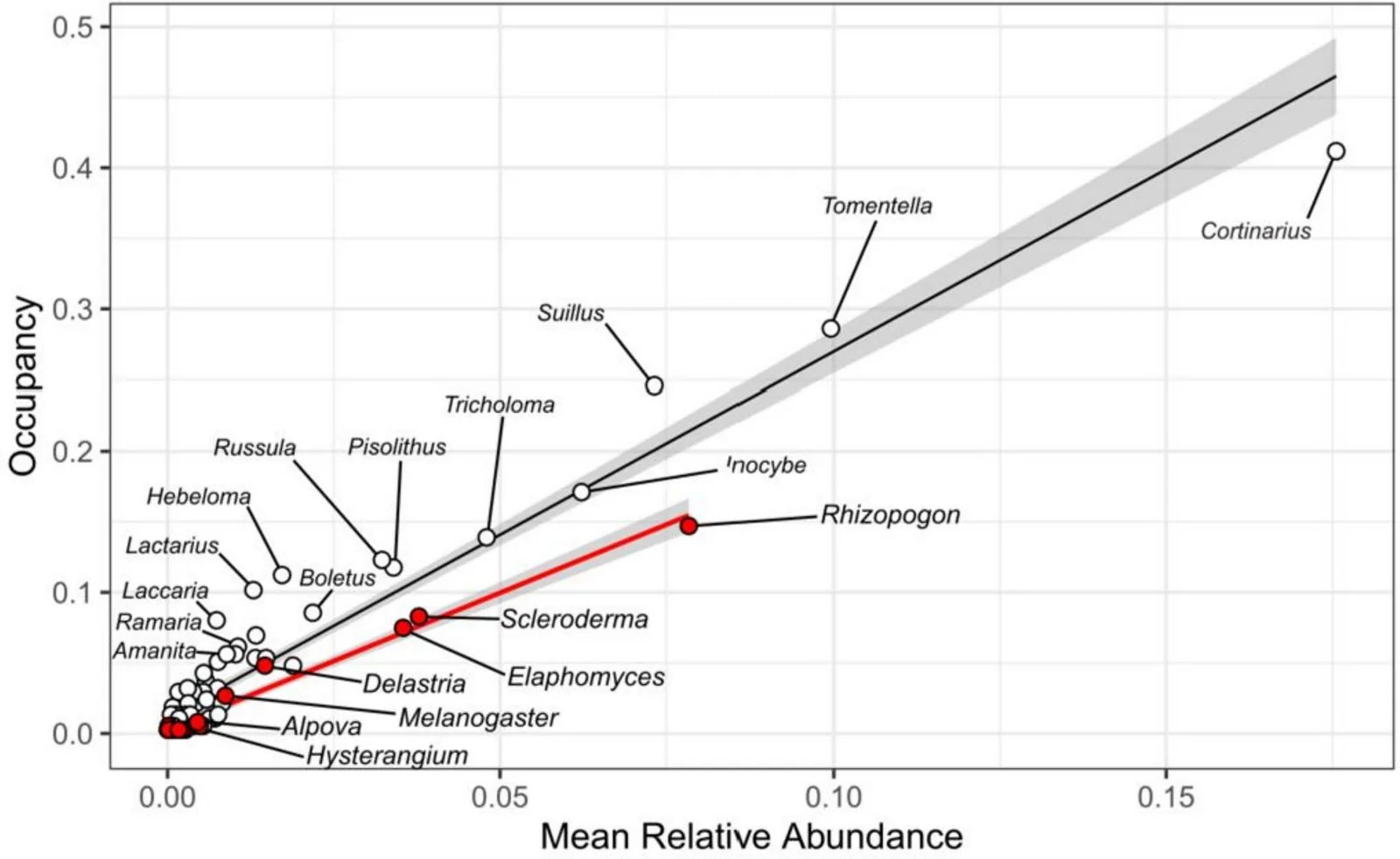
Comparison of the occupancy versus relative abundance of epigeous (white) and hypogeous (red) ectomycorrhizal (EM) fungal genera in the spore trap samples across sites. Occupancy represents the proportion of samples in which the EM genus was found out of the total number of spore trap samples. Relative abundances are based on the mean number of sequence reads per EM fungal genus across all samples. Black and red lines of best fit are based on simple linear regression. The grey shading around each line represents 95% confidence intervals

In the spore trap samples, hypogeous EM genera ranged in abundance (mean number of sequences per sample) and occupancy (number of samples present) across sites (Fig. 2). Hypogeous EM genera had a significantly lower occupancy than that of epigeous EM genera at a given abundance (p < 0.001), suggesting dispersion events were more episodic.

At the site level, hypogeous EM genera were among the ten most abundant EM genera at seven of the eight sites and the most abundant EM genus at three sites (GRTL, *Elaphomyces*; PNW, *Rhizopogon*; DSW, *Rhizopogon*) (Table 1).

**Table 1:** Rank abundance of epigeous and hypogeous ectomycorrhizal (EM) fungal genera across the eight study sites. Rank is based on relative abundance of sequence reads of each genus compared to all EM fungal reads at each site. In parentheses is abundance, followed by occupancy (proportion of samples present in) of each genus. Hypogeous EM genera are noted in bold. The number in parentheses next to each site name is the number of spore trap samples

Rank	SE (107)	PNW (81)	GRTL (59)	PRAI (58)	DSW (24)	SROC (20)	NE (13)	TAIG (12)
1	*Cortinarius* (0.39, 0.57)	***Rhizopogon*** (0.66, 0.25)	***Elaphomyces*** (0.19, 0.07)	*Tomentella* (0.22, 0.28)	***Rhizopogon*** (0.23, 0.29)	*Cortinarius* (0.21, 0.40)	*Cortinarius* (0.25, 0.92)	*Cortinarius* (0.36, 0.83)
2	*Tricholoma* (0.18, 0.30)	***Hysterangium*** (0.06, 0.01)	*Suillus* (0.18, 0.29)	***Pachyphlodes*** (0.13, 0.03)	*Cortinarius* (0.18, 0.29)	*Suillus* (0.17, 0.55)	*Suillus* (0.14, 0.31)	***Alpova*** (0.09, 0.25)
3	***Rhizopogon*** (0.06, 0.14)	*Cortinarius* (0.04, 0.35)	*Russula* (0.15, 0.22)	***Scleroderma*** (0.13, 0.16)	*Suillus* (0.16, 0.08)	*Boletus* (0.09, 0.25)	*Tomentella* (0.12, 0.62)	*Alnicola* (0.08, 0.42)
4	*Suillus* (0.06, 0.33)	*Inocybe* (0.03, 0.33)	*Cortinarius* (0.13, 0.36)	*Cortinarius* (0.05, 0.12)	*Sebacina* (0.12, 0.04)	*Hebeloma* (0.08, 0.35)	*Russula* (0.10, 0.38)	*Lactarius* (0.08, 0.67)
5	*Tomentella* (0.05, 0.45)	*Astraeus* (0.02, 0.10)	*Strobilomyces* (0.05, 0.12)	***Elaphomyces*** (0.05, 0.10)	*Astraeus* (0.04, 0.12)	***Rhizopogon*** (0.08, 0.25)	*Lactarius* (0.07, 0.31)	*Paxillus* (0.08, 0.58)
6	*Pisolithus* (0.03, 0.30)	*Piloderma* (0.02, 0.19)	*Boletus* (0.03, 0.10)	*Inocybe* (0.05, 0.10)	*Pisolithus* (0.04, 0.12)	***Hysterangium*** (0.04, 0.05)	*Amanita* (0.06, 0.23)	*Thelephora* (0.08, 0.58)
7	*Hebeloma* (0.02, 0.15)	*Suillus* (0.02, 0.20)	*Fuscoboletinus* (0.03, 0.08)	*Caloboletus* (0.04, 0.05)	*Tomentella* (0.03, 0.12)	*Paxillus* (0.04, 0.25)	*Inocybe* (0.03, 0.38)	*Suillus* (0.06, 0.50)
8	*Ramaria* (0.02, 0.16)	*Tomentella* (0.02, 0.23)	*Hydnellum* (0.02, 0.10)	*Membranomyces* (0.04, 0.03)	*Hebeloma* (0.02, 0.04)	*Russula* (0.04, 0.30)	*Tylopilus* (0.03, 0.31)	*Hebeloma* (0.05, 0.58)
9	*Amanita* (0.01, 0.11)	*Tricholoma* (0.02, 0.09)	*Lactarius* (0.02, 0.05)	*Fuscoboletinus* (0.03, 0.05)	*Inocybe* (0.02, 0.12)	*Tricholoma* (0.04, 0.15)	*Xanthoconium* (0.03, 0.15)	*Leccinum* (0.04, 0.42)
10	*Amphinema* (0.01, 0.09)	***Delastria*** (0.01, 0.02)	***Scleroderma*** (0.02, 0.12)	***Melanogaster*** (0.03, 0.05)	*Tricholoma* (0.02, 0.12)	*Lactarius* (0.03, 0.20)	*Boletus* (0.02, 0.31)	*Amphinema* (0.01, 0.33)

**Fig. 3:**
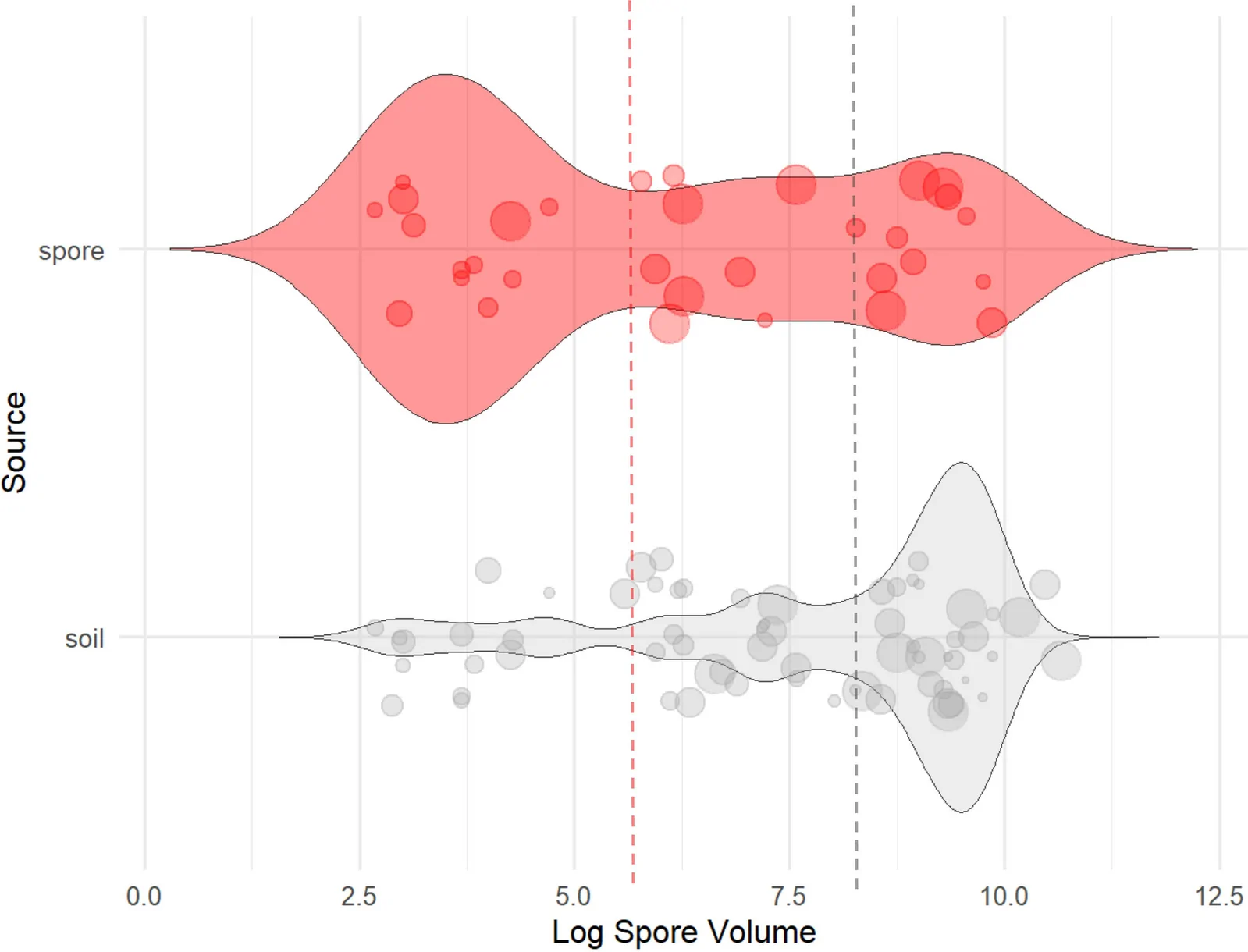
Spore volumes (log) of hypogeous ectomycorrhizal fungi sampled in spore traps (red) and soils (grey) across sites. Dashed vertical lines represent the mean spore volume of each sample source. The width of the violin plots is scaled to the relative abundances of the OTUs sampled. Circles represent individual OTUs, with the circle size representing the mean relative abundance of that OTU within each dataset

In terms of occupancy, EM hypogeous genera were among the 10 most frequent EM genera at seven sites, but the most frequent genus at only one of the sites (DSW). With respect to specific genera, *Rhizopogon* was the most abundant and frequently occurring hypogeous EM genus, being among the three most abundant and the fifth most frequent EM genera across all sites (Fig. 2). Notably, however, this genus was functionally absent from the Alaska site (TAIG), which was dominated by *Picea* (not considered a typical *Rhizopogon* host). Other dominant hypogeous EM genera represented a diverse range of both Ascomycota (*Elaphomyces*, *Genea*, *Pachyphlodes*) and Basidiomycota (*Alpova*, *Gautieria*, *Hysterangium*, *Melanogaster*, *Truncocolumella*) lineages. Comparing the mean spore volumes of hypogeous EM taxa present in spore traps versus those present in soils, those in spore traps had significantly smaller mean volumes (Fig. 3; p < 0.001).

Despite having 500 µm mesh to prevent debris and insects from entering the spore traps, particulates were present on some of the filters (Figure S3). While the particulates appeared to largely be ash from controlled burns, they could also represent soil contamination, both of which may have carried EM fungal propagules into the traps rather than the propagules arriving independently via air. The abundances of both hypogeous and epigeous EM sequence reads were not, however, significantly related to filter particulate abundance (Figure S4; p > 0.6). Samples with higher richness and abundance of epigeous EM OTUs often also had lower richness and abundance of hypogeous EM OTUs and vice versa (Figure S5). These results, in conjunction with the use of landscape fabric under the traps (Fig. 1), suggest that soil contamination into traps was not likely responsible for the presence or abundance of hypogeous EM spores in the spore traps.

**Fig. 4:**
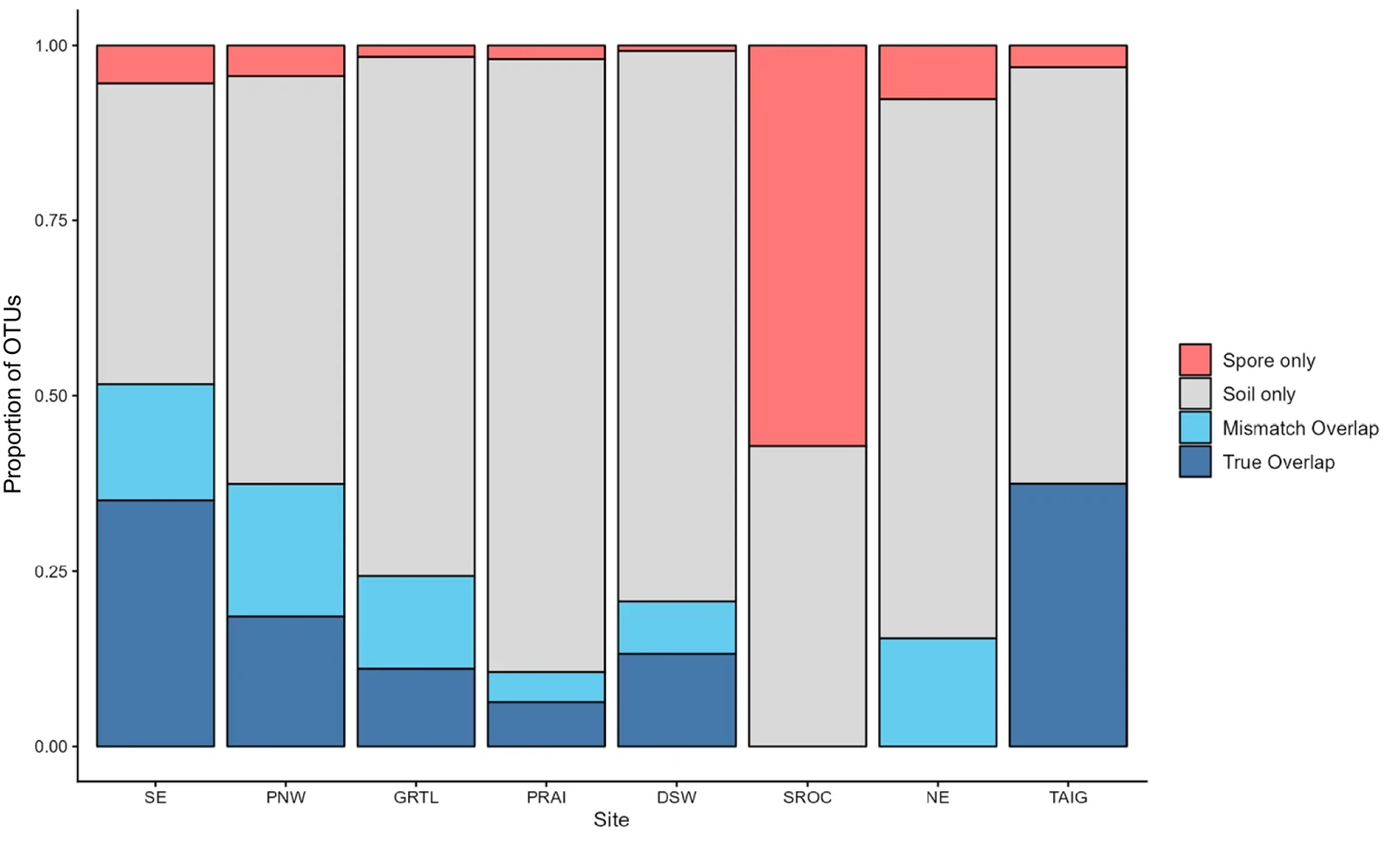
Overlap in composition of hypogeous ectomycorrhizal fungi between spore trap and soil samples by site. Categories scaled by proportion: Spore only = OTUs only found in spore dataset. Soil only = OTUs only found in the soil dataset. True overlap = OTUS found in both spore trap and soil samples in the same habitat. Mismatch overlap = OTUs found in both spore trap and soil samples, but not in the same habitat

Comparing the hypogeous EM OTUs present in the spore trap and soil samples revealed that 45 OTUs (4%) were only present in the spore trap samples, 792 OTUs (68%) were only present in the soil samples, and 333 OTUs (28%) were present in both sample types (Table S4). The proportion of hypogeous EM OTUs found in the spore traps only, soil samples only, or in both sample types varied by site (Fig. 4). Two of the sites, SROC and NE, had no hypogeous EM OTUs that overlapped between the spore trap and soil samples, while the other six sites varied widely in the proportion of overlapping OTUs. For those present in both sample types, many were present in the same habitat (referred to as ‘true overlap’). However, 18 hypogeous EM OTUs were found in different habitats and sample types at the same site (referred to as ‘mismatched overlap’).

For example, at the GRTL site, *Elaphomyces anthracinus* was sampled in the soil in the oak forest as well as in a spore trap in the grassland, which were located ~ 400 m apart. Similarly, at the SE site, *E. cyanosporus* was sampled in the soil in the oak forest as well as in a spore trap in the pine forest, which were located ~ 1.5 km apart. Additionally, eight hypogeous OTUs were found in both the spore trap and soil samples, but at different sites (referred to as ‘mismatched overlap’). For example, although present in low abundance (sequence read counts < 5), *Hydnotrya* sp. was sampled in the soils of all habitats at the GRTL site as well as in an oak forest spore trap at the NE site.

## Discussion

Contrary to our conventional understanding about hypogeous EM fungi dispersal, we found that the aerial EM mycobiome across eight sites in the United States contained a consistent and diverse range of hypogeous EM fungi taxa. Relative to epigeous EM fungi, the patterning of hypogeous EM fungi in the spore trap samples was generally more episodic, i.e. having lower frequency and higher abundance when present. This pattern likely reflects distinct biological events that expose hypogeous EM sporocarps to wind, rather than the more continuous entry in air of epigeous EM spore dispersal. This pattern was also not likely driven by soil contamination as shown by a lack of correlation between filter particulate load and hypogeous EM OTU abundance. Notably, the hypogeous EM taxa encountered in the spore trap samples had significantly smaller volume spores on average than those found only in soil, suggesting specific traits can facilitate their inclusion in the aerial EM mycobiome. While observing the same hypogeous EM OTUs in spore trap and soil samples within the same habitat is consistent with local-scale dispersal, we also found multiple instances of the same hypogeous EM OTUs being present in different sample types across habitats within a site as well as among sites. These latter findings, although not common, suggest that aerial dispersal may also facilitate longer distance dispersal of hypogeous EM fungi in the same way it does for epigeous EM fungi (Chaudary et al. 2022). Together, these findings indicate that the soil-atmosphere boundary is more porous for hypogeous EM fungi than previously recognized, positioning wind as a secondary but ecologically relevant pathway that complements mammalian-mediated dispersal.

The movement of hypogeous EM spores into air likely occurs through multiple non-mutually exclusive pathways. We divide these into three primary categories; 1) production of mitosporic mats and semi-hypogeous sporocarps, 2) disturbance to soil that exposes hypogeous sporocarps to air, and 3) movement of hypogeous sporocarps and associated spores in and on animals to alternate locations. Regarding mitosporic mats, Healy et al. (2013) demonstrated that these mats represent the anamorphic stage of many truffle-forming Ascomycota in the order Pezizales (e.g. *Hydnotyra*, *Hydnobolites, Pachyphlodes*, and *Tuber*). While the morphology of mitospores and ascospores are different (Healy et al. 2013), our analyses, which only used DNA-based identification, cannot distinguish which spore type was present in our samples. We therefore consider taxa capable of forming these mats to potentially be ‘false positives’ for aerial hypogeous EM dispersal but note that exclusion of genera with these characteristics (which are all Pezizales fungi) does not strongly alter our overall conclusions due to their low occurrence and abundance within spore trap samples. Some truffle-forming EM fungal genera also locate their sporocarps at the soil-air interface, a phenomenon referred to as semi-hypogeous (Watling 2006). A prominent example is the genus *Scleroderma*, which has species that produce sporocarps at a range of locations; hypogeous (*S. patagonicum*, Nouhra et al. 2012), semi-hypogeous (e.g. *S. bermudense*, Séne et al. 2018), or epigeous (*S. areolatum*, Kuo and Methven 2014). Interestingly, at the GRTL site, *S. citrinum* sporocarps are frequently encountered tens of centimeters above the soil on the surfaces of decomposing logs (P. Kennedy, personal observation). By locating their sporocarps at or above the soil surface and therefore directly exposed to wind, it is thus not unsurprising that this genus or others with semi-hypogeous sporocarps (e.g. *Sclerogaster*) were present in the aerial EM mycobiome.

The second category by which hypogeous EM fungi may enter the aerial EM mycobiome is through soil disturbance. This can take many forms: freeze/thaw events, animal trampling/scratching, or animal digging – all of which open or remove soil around truffles and expose them directly to air–soil interface (Caiafa et al. 2021; Elliott et al. 2022). Once exposed, rupture of the truffle peridium (i.e. the outer surface enclosing the spores) can also occur via many mechanisms: incomplete animal consumption, insect emergence, or abiotic weathering, thus allowing spores to be carried by wind or splash into air (Elliott et al. 2022). This category is also consistent with the documentation of AM fungal spores likely entering air via anthropogenic soil disturbance (Chaudhary et al. 2020), although the boundary layer present at the forest air–soil interface likely acts as an important barrier, particularly for species with larger diameter spores that probably require major weather events to become and stay aerosolized.

The third category of entry into the aerial EM mycobiome is movement of hypogeous EM sporocarps or spores out of the soil. As noted above, many animals remove hypogeous (and epigeous) EM fungi from the soil and cache them in alternate locations for later consumption (Elliott et al. 2022). Elevated caching behavior, particularly moving EM sporocarps in various locations in trees, appears to be relatively common among small arboreal mammals, such as squirrels (Maser et al. 2008) and hypogeous EM sporocarps can make a significant portion of the small mammal diet seasonally (North et al. 1997). Consumption in elevated positions would likely facilitate spore release into air if sporocarps are left partially consumed (exposing spores to wind) or parts of the sporocarp with adhering spores are discarded during that process. Additionally, hypogeous EM spores can enter the air secondarily following animal defecation onto the soil surface. Animal feces can be a concentrated source of hypogeous EM spores (Borgmann-Winter et al. 2023) and its subsequent breakdown at the soil surface may also liberate spores into air. A final animal-mediated vector is the attachment of hypogeous EM spores to the bodies of animals and their subsequent release during movement. This phenomenon has been well documented in birds (Elliott el. 2019; Caiafa 2025), although may apply to many other animals (Ori et al. 2021), including insects (Elliott et al. 2022) that are also known to disperse EM spores (Thomas and Thomas 2022).

It is also worth noting that some EM hypogeous fungi have been shown to have incredibly tough spores that can persist and remain viable in soil following extreme stressors (Izzo et al. 2006) and for long periods of time (Bruns et al. 2009; Nguyen et al. 2012; Shemesh et al. 2023), which may be an important factor for some genera in our dataset (e.g. *Rhizopogon*). Additionally, recent studies have shown that hypogeous EM spores remain intact and putatively viable after passage through the guts of herbivores and then predators (Stephens et al. 2025). These traits suggest that the hypogeous EM fungi have high longevity/stress tolerance compared to spores of some other types of fungi, potentially giving them greater opportunity to be carried by wind because of their persistence. Finally, two of the hypogeous EM genera in our dataset, *Scleroderma* and *Elaphomyces*, produce highly hydrophobic spores that make their gleba very powdery and thus easy to aerosolize. This characteristic, along with their relatively small spore volumes, likely enhances their capacity to enter air, and may help explain their relatively high abundance in spore traps at two of the study sites (Table 1). Supporting this possibility, Reynolds (2011) showed experimentally that *Elaphomyces* has a high capacity for passive wind dispersal.

Looking forward, we envision several opportunities to refine knowledge about hypogeous EM aerial dispersal. While our results consistently showed a presence of hypogeous EM fungi in the air, which have also been observed in previous passive trap studies (Peay et al. 2012; Borgmann-Winter et al. 2023), this signal is largely absent in other recent global aerobiome surveys (e.g., Tipton et al. 2019; Abrego et al. 2024). This discrepancy suggests that passive traps, which rely on wind and rain deposition rather than active suction, capture a somewhat different suite of the aerial mycobiome (Minahan et al. 2024; Schlegel et al. 2024). As such, additional research directly comparing aerial spore sampling methods would help better determine the extent to which they capture similar communities. It would also be beneficial to include direct observations of spores in addition to molecular analyses (i.e. Stephens et al. 2025), as the latter is an indirect measure of abundances that can be skewed by factors such as differences in gene target copy number (Lofgren et al. 2019). Furthermore, while our OTU-based approach identified the potential for broad movement patterns, tracking true ‘source-sink’ dynamics from soil to air will require higher-resolution molecular analyses (e.g., SNPs) to distinguish specific hypogeous EM genotypes. We also acknowledge that our monthly sampling intervals likely obscure the precise triggers of the pulsed abundances that we observed of hypogeous EM fungi. Finally, we reiterate that we consider wind as a secondary, rather than dominant, dispersal mechanism compared to mammalian mycophagy. However, by documenting the presence of this overlooked pathway at a continental scale, we hope to broaden the recognition of alternative dispersal modes and encourage a more integrated view of the hypogeous EM life cycle.

## Supplementary Information

Below is the link to the electronic supplementary material.

Supplementary file1 (PDF 359 KB)

Supplementary file2 (PDF 95 KB)

Supplementary file3 (PDF 82 KB)

Supplementary file4 (PDF 143 KB)

Supplementary file5 (XLSX 15 KB)

Supplementary file6 (XLSX 22 KB)

Supplementary file7 (XLSX 15 KB)

Supplementary file8 (XLSX 11 KB)

## Data Availability

Raw .fastq sequence files were deposited in the National Center for Biotechnology Information (NCBI) Short Read Archive (SRA) under BioProject numbers PRJNA1291903 (spore trap) and PRJNA1327291 (soil). The authors declare no competing interests.
