# Supplementary figures and images for: Is aerial dispersal an overlooked pathway for ectomycorrhizal truffle fungi?
Source: Mycorrhiza. 2026 Jul 28;36(4):53. doi: 10.1007/s00572-026-01296-x (PMC13407476; doi:10.1007/s00572-026-01296-x)

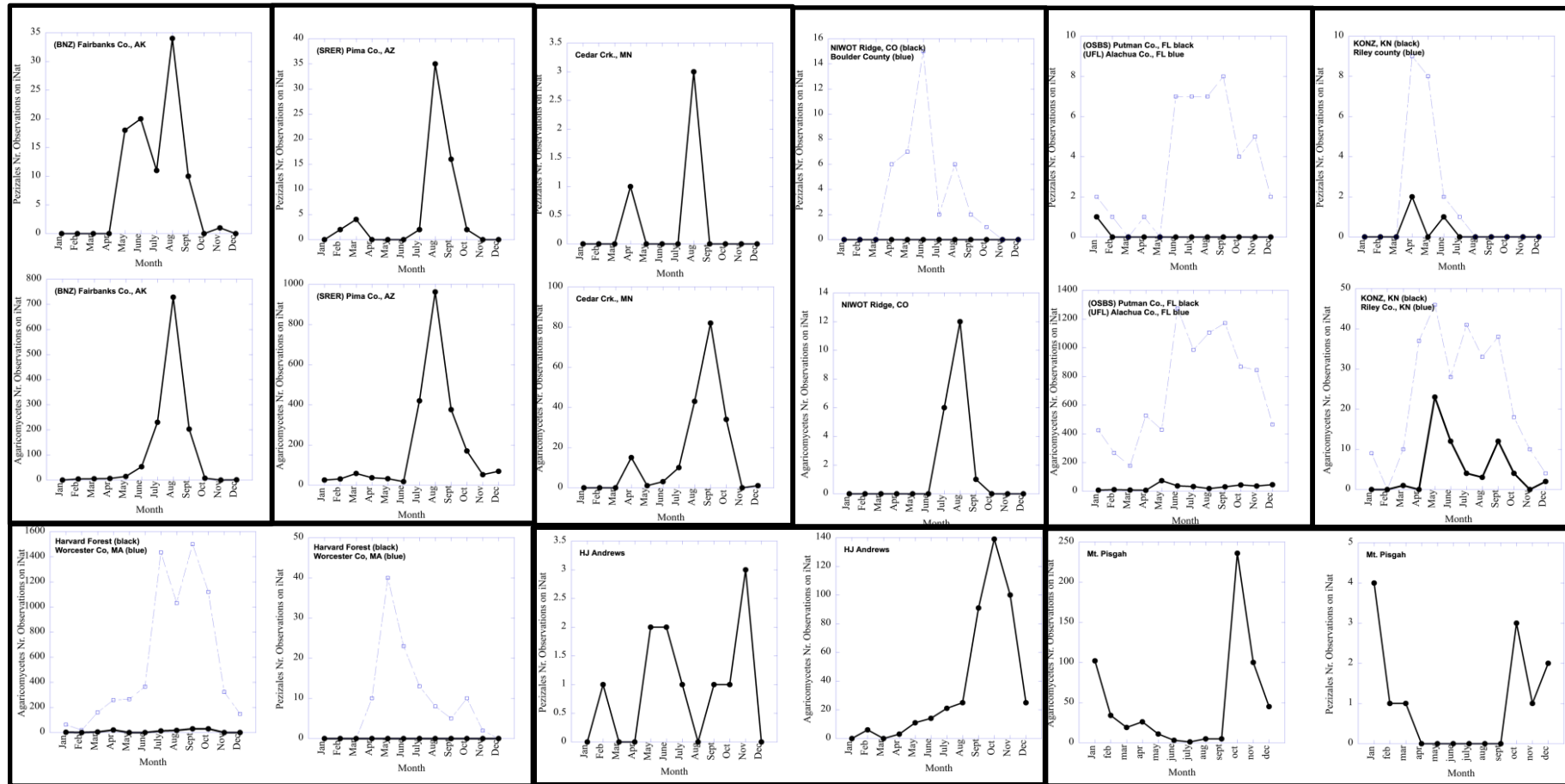

Supplement: Supplementary file 1 — Supplementary file1 (PDF 359 KB) [file 572_2026_1296_MOESM1_ESM.pdf]

## Spore Trap

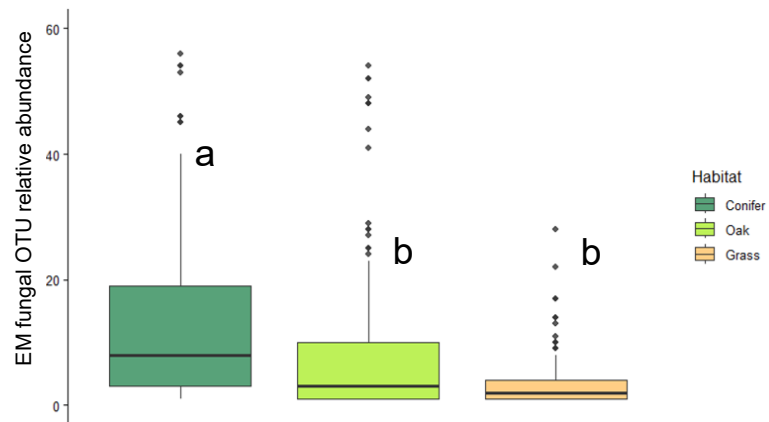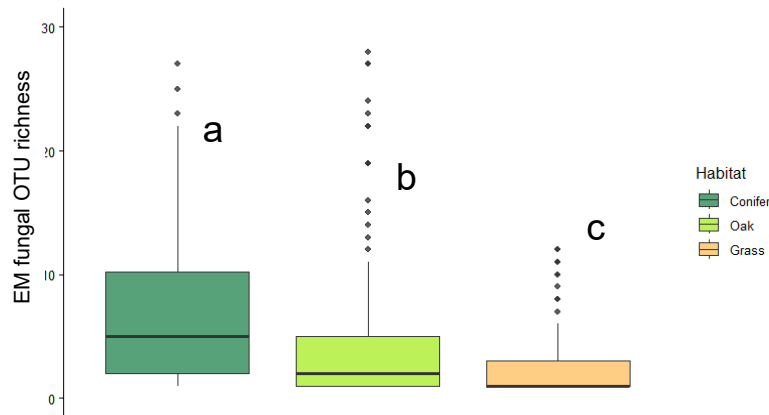

## Soil

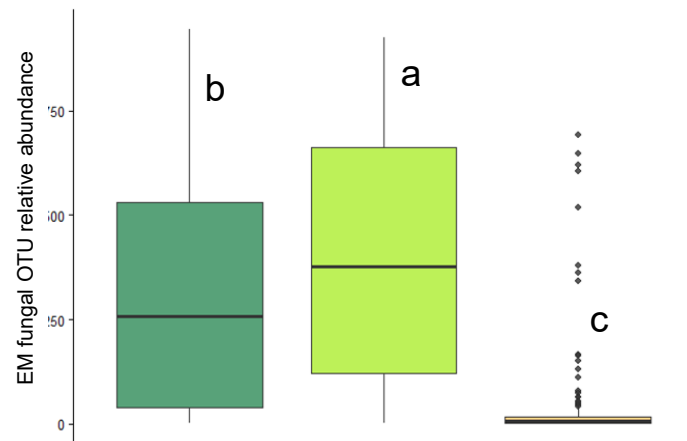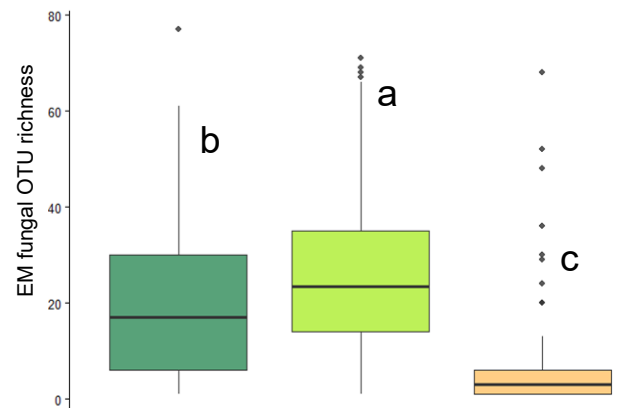

Supplement: Supplementary file 2 — Supplementary file2 (PDF 95 KB) [file 572_2026_1296_MOESM2_ESM.pdf]

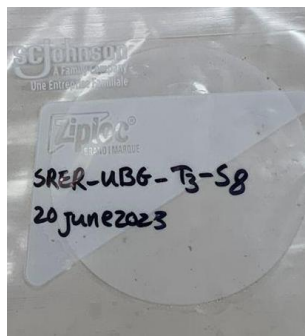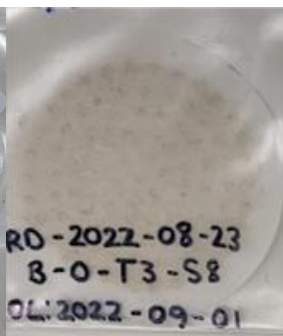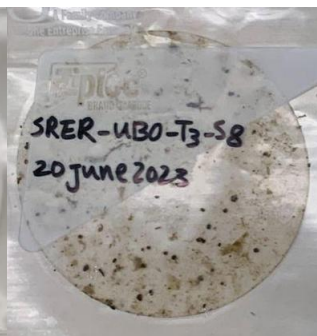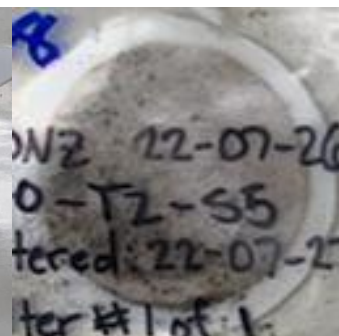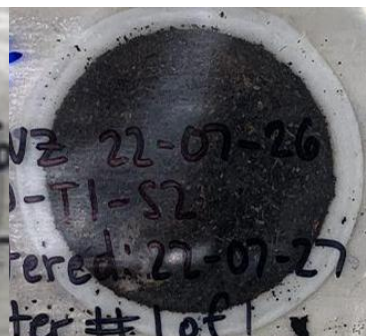

Supplement: Supplementary file 3 — Supplementary file3 (PDF 82 KB) [file 572_2026_1296_MOESM3_ESM.pdf]

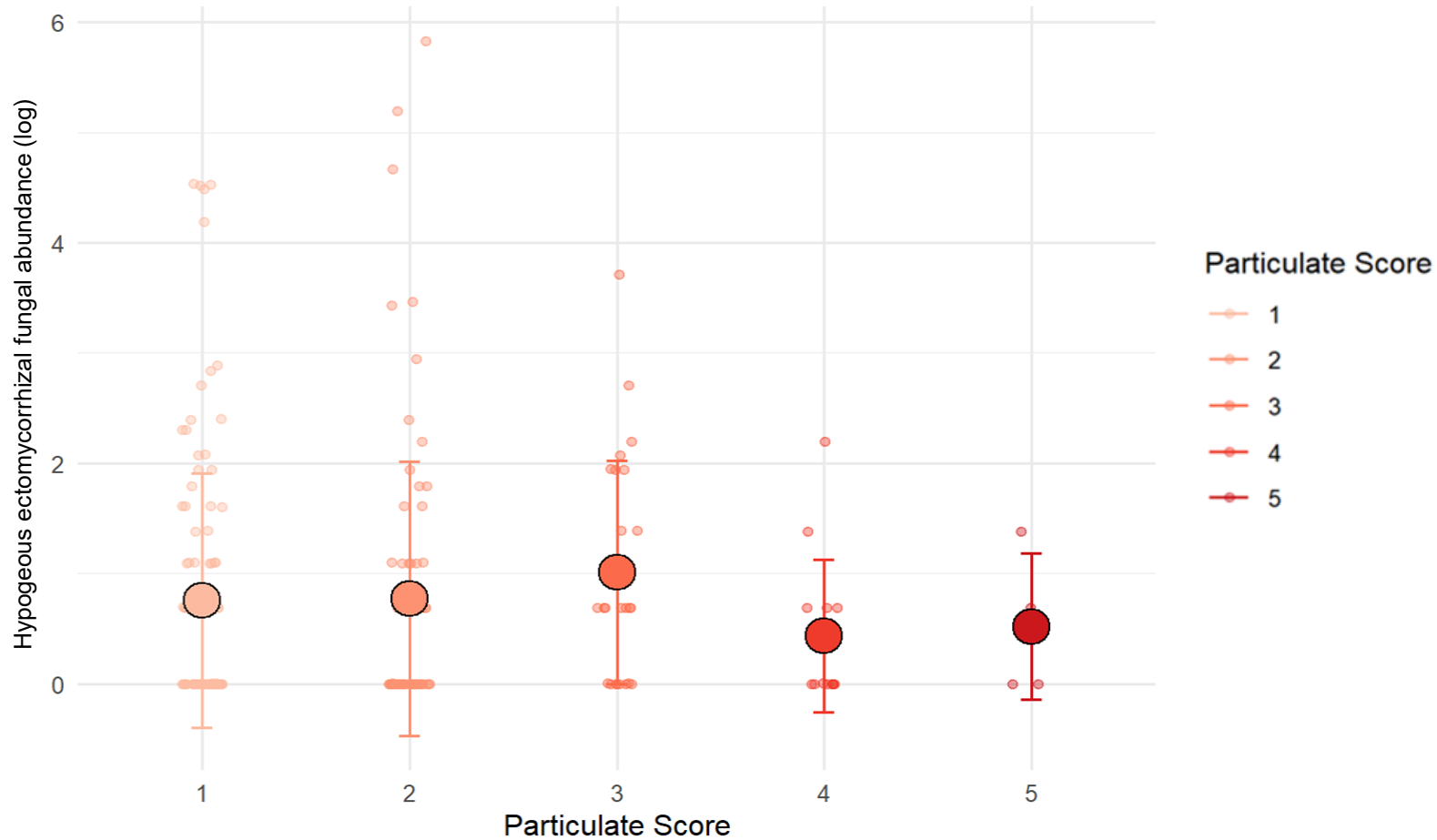

Supplement: Supplementary file 4 — Supplementary file4 (PDF 143 KB) [file 572_2026_1296_MOESM4_ESM.pdf]
